# Current Status of the Cognitive Impairment in Chronic Kidney Disease

**DOI:** 10.1298/ptr.R0033

**Published:** 2024-11-06

**Authors:** Yuhei OTOBE

**Affiliations:** 1 Graduate School of Rehabilitation Science, Osaka Metropolitan University, Japan

**Keywords:** Chronic kidney disease, Dialysis, Cognitive impairment, Dementia, Physical exercise

## Abstract

Chronic kidney disease (CKD) is a recognized risk factor for cognitive impairment and dementia. Unfortunately, the number of patients with both CKD and dementia has been steadily increasing with the aging patient population. Therapeutic management and clinical decision-making become more challenging in patients with dementia who often experience worsening prognoses, highlighting the urgency of developing effective countermeasures. This review explores available research on the epidemiology, contributing factors, mechanisms, and outcomes of cognitive impairment and dementia in patients with CKD while outlining the impact of exercise therapy on cognitive function.

## Introduction

A rapid increase in the older population worldwide has turned the spotlight on the aging of patients with chronic kidney disease (CKD) and those undergoing dialysis. This phenomenon has promoted an increase in the number of patients with cognitive impairment^1)^, leading to reports of various problems such as poor prognosis, increased caregiving need, and withdrawal of dialysis. Therefore, measures that address this issue are urgently needed. However, effective treatment options for cognitive impairment and dementia remain unclear, highlighting the need for evaluation and managing risk factors to prevent cognitive decline. This review explores the epidemiology, adverse effects, and pathophysiology of cognitive impairment and dementia in patients with CKD while also outlining the role of renal rehabilitation in the prevention of cognitive decline.

## Epidemiology of Cognitive Impairment and Dementia

### Hemodialysis patients

Evidence has shown a high prevalence of dementia and cognitive impairment among dialysis patients. Among the 327336 hemodialysis patients in Japan, 6.7% of those aged 65–74 years and 24.3% of those aged ≥75 years have dementia^1)^. Furthermore, among 338 hemodialysis patients aged 55 years and older in the United States, 13.9%, 36.1%, and 37.3% had mild, moderate, and severe cognitive impairment, whereas only 12.7% had normal cognitive function^2)^. Despite the inconsistent methods used for evaluating dementia and cognitive impairment, available evidence clearly shows that dialysis patients are at high risk for dementia.

### Pre-dialysis patients with CKD

Studies have also reported a high prevalence of dementia and cognitive impairment even among pre-dialysis patients with CKD. Indeed, a study involving 23405 community-dwelling adults aged 45 years and older in the US (mean age, 64.9 years, with 11% being patients with CKD) found that patients with CKD had a higher risk of cognitive impairment than patients with no CKD. The same study showed that for every 10 mL/min/1.73 m^2^ decrease in estimated glomerular filtration rate (eGFR), the risk of cognitive impairment increased by 11% and 17% among those under 65 years old and those aged 65 years and older, respectively^3)^.

Furthermore, patients with CKD have a higher risk of newly developing dementia. In fact, one study that investigated the new onset of cognitive impairment among 3679 community-dwelling adults over a 2-year follow-up period found that the incidence rates of new cognitive impairment were 5.8%, 9.9%, and 21.5% in those with a baseline creatinine clearance (Ccr) of ≥60, 45–59, and <45 mL/min/1.73 m^2^, respectively. Moreover, the same study^4)^ found that the risk of new cognitive impairment in the group with a Ccr of <45 mL/min/1.73 m^2^ was 2.14 times higher than that in the group with a Ccr of ≥60 mL/min/1.73 m^2^. Another study found that not only the baseline level of kidney function but also longitudinal changes therein affect the incidence of dementia and cognitive impairment, with a 20% decrease in eGFR per year being associated with a 1.36 times increase in the risk for subsequent cognitive decline^5)^. Moreover, patients who exhibited an annual decline in eGFR of ≥4 mL/min/1.73 m^2^ had a 5.35 times higher risk for developing vascular dementia (VaD) over 7 years than those with a lesser decline^6)^. Therefore, treatments that delay the decline in kidney function may be among the important strategies for preventing cognitive decline and dementia.

## Adverse Effects of Cognitive Impairment in Patients with CKD

**Fig. 1.**
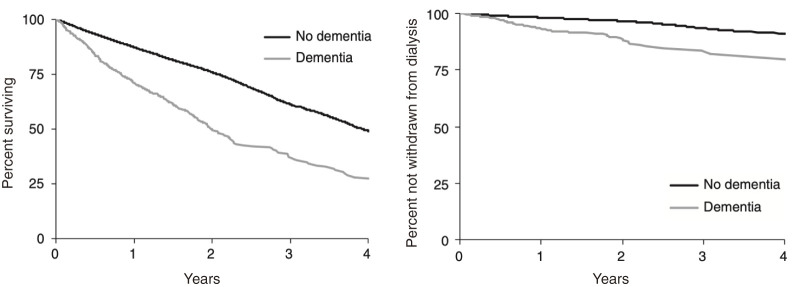
Association between dementia and risk of death and dialysis withdrawal^7)^

Dementia and cognitive impairment significantly impact the prognosis of patients with CKD. In fact, the Dialysis Outcomes and Practice Pattern Study (DOPPS), which included 16694 hemodialysis patients from 7 countries (United States, France, Japan, Spain, Italy, Germany, and the United Kingdom), reported that patients with dementia had a higher overall mortality than did those without dementia (Fig. 1)^7)^. Notably, the risk for overall mortality was approximately 1.2 times higher for the entire cohort and as much as 2.1 times higher for Japanese patients specifically^7)^. Such a risk has also been observed in pre-dialysis patients with CKD as reflected in a study that found an association between a decrease in cognitive scores and increased risk of mortality among pre-dialysis patients with CKD^8)^. Furthermore, another study found that each 1-point increase in the Mini-Mental State Examination score among pre-dialysis patients with CKD aged 80 years and older was associated with a 29% reduction in the risk of all-cause mortality and a 39% reduction in cardiovascular mortality within 3 years^9)^. These findings indicate that the coexistence of demen-tia and cognitive impairment is a significant barrier to longev-ity in both dialysis and non-dialysis patients with CKD.

The coexistence of dementia also influences decisions regarding dialysis withdrawal. A study conducted in Japan found that among cases who died following withdrawal of hemodialysis, 93.9% were elderly patients aged 65 years and older, a high proportion of whom (66.3%) had cognitive impairment^10)^. Additionally, the DOPPS showed that patients with dementia had a 1.6 times higher risk for dialysis withdrawal than those without dementia (Fig. 1)^7)^. Several factors influence the decision to withdraw from dialysis, including deterioration of overall health, discomfort associated with dialysis, and a decline in physical function. Therefore, dialysis is not withdrawn based solely on the presence of dementia. However, dementia is evidently a significant factor in the decision to withdraw from dialysis. This underscores the increasing importance of developing systems for advanced care planning and shared decision-making, as well as providing palliative care after dialysis withdrawal.

## Risk Factors of Cognitive Impairment

Numerous studies are available on the risk factors for dementia onset and intervention strategies in patients with no CKD and the general population. Accordingly, various risk factors for the onset of dementia have been identified, among which 45% are potentially modifiable^11)^. Potentially modifiable factors are categorized across all life stages, including early life, midlife, and late life. Early life risk factors include poor education. Meanwhile, midlife risk factors include hearing loss, high low-density lipoprotein cholesterol, depression, traumatic brain injury, physical inactivity, diabetes mellitus, smoking, hypertension, obesity, and excessive alcohol intake. Finally, late-life risk factors include social isolation, air pollution, and untreated visual loss^11)^. As described earlier, many of the potentially modifiable risk factors are related to lifestyle and lifestyle-related diseases. Therefore, comprehensive intervention, including the management and treatment of CKD, can contribute to the prevention of dementia.

## Specific Risk Factors of Cognitive Impairment in CKD

**Fig. 2.**
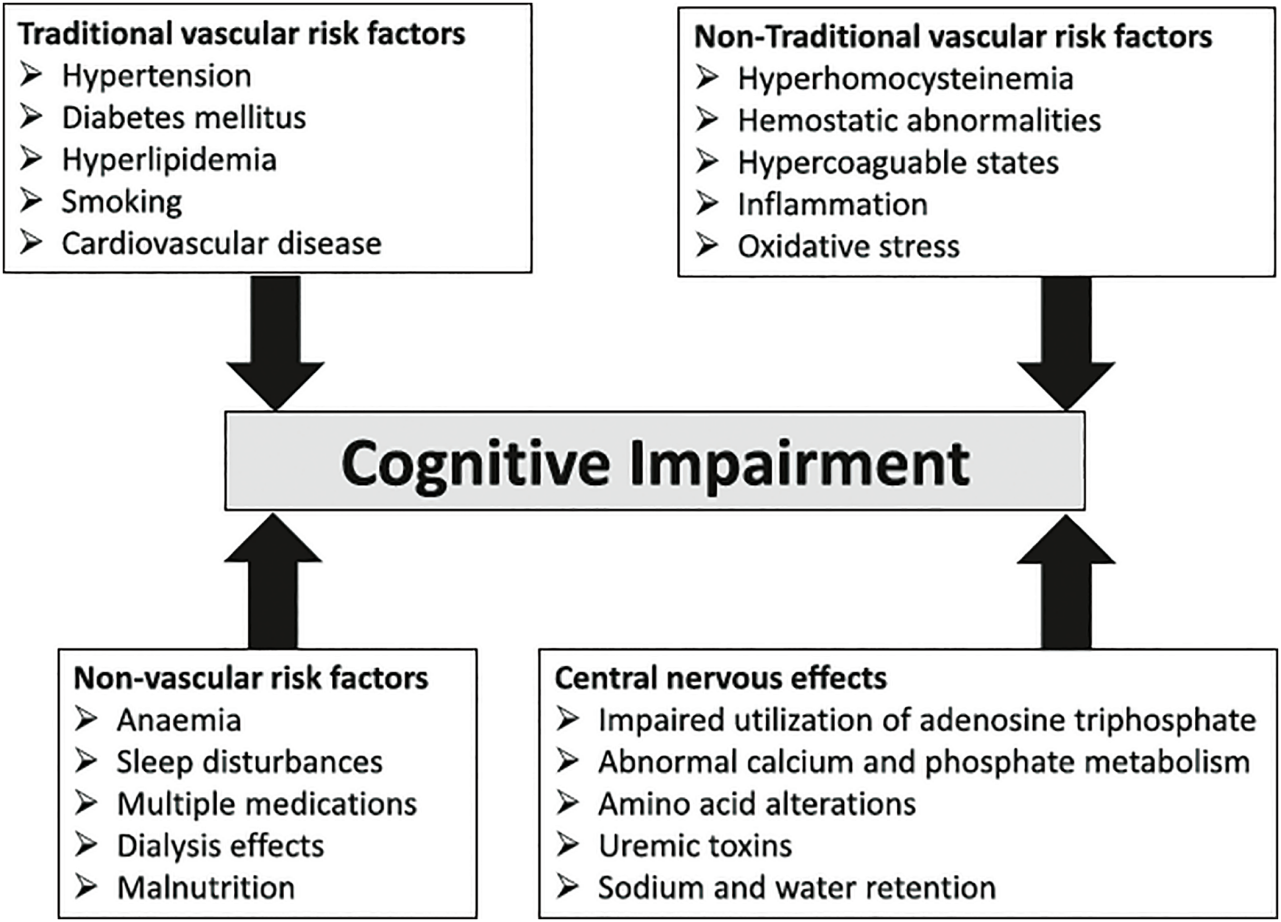
Pathophysiology of cognitive decline in patients with chronic kidney disease Modified from reference^12)^.

Various specific risk factors for the development of dementia and cognitive impairment in patients with CKD have also been reported. For example, Etgen^12)^ categorized these risk factors into 4 main groups: (1) traditional vascular risk factors, such as diabetes mellitus, hypertension, and dyslipidemia; (2) non-traditional vascular risk factors, such as chronic inflammation and oxidative stress; (3) non-vascular risk factors, such as anemia and malnutrition; and (4) central nervous system factors, such as uremic toxins (Fig. 2). These factors may cause vascular damage, endothelial dysfunction, and neurotoxicity. Furthermore, the accumulation of uremic toxins due to worsening kidney function has been hypothesized to adversely affect the central nervous system. Additionally, recent reports suggest that hemodialysis treatment itself may induce focal cerebral ischemia, potentially leading to the development of dementia in the long term^13)^. These risk factors do not individually cause cognitive decline but rather form interrelationships of multiple factors that exert a negative impact. However, a clear understanding of such interrelationships has yet to be reached, warranting further accumulation of evidence.

## Mild Cognitive Impairment

In recent years, patients with mild cognitive impairment (MCI) have gained attention as a target population for dementia prevention. MCI refers to a condition characterized by a decline in cognitive function, including memory, which does not meet the criteria for dementia. The diagnostic criteria for MCI include (1) complaints of cognitive decline, (2) cognitive impairment abnormal for the individual’s age but does not meet the criteria for dementia, and (3) the ability to perform basic activities of daily living independently^14)^. Owing to these characteristics, MCI has been considered an intermediate stage between normal aging and dementia. MCI has a high conversion rate to dementia, with 5%–10% of individuals having MCI developing dementia each year as opposed to only 1%–2% of non-MCI individuals^15)^. However, the reversion rate from MCI to a normal cognitive state is also high, with 14%–44% of individuals having MCI later showing improvement to normal cognitive function^16–19)^. Therefore, identifying individuals with MCI and implementing multifactorial interventions, such as physical exercise and lifestyle modification, are imperative.

Evidence suggests that the prevalence of MCI is higher in CKD patients than in patients with no CKD. Indeed, our previous study showed that 62.5% of older patients with pre-dialysis CKD had MCI based on the Japanese version of the Montreal Cognitive Assessment, a screening tool for MCI^20)^. Additionally, one study reported that 29.0%–33.7% of patients with CKD had MCI^21)^, whereas another reported that the prevalence of MCI among community-dwelling older adults in Japan was 18.8%^22)^. These studies highlight the relatively higher prevalence of MCI in patients with CKD. Currently, only a few studies specifically present the conversion rate to dementia or examine intervention strategies in patients with CKD with MCI. However, patients with CKD with MCI may also have a high reversion rate to normal cognitive function, similar to the community-dwelling population. Therefore, these patients constitute a promising target population for intensive dementia prevention efforts.

## Association Between Physical Function and Cognitive Impairment

Physical and cognitive functions mutually influence each other, with gait ability, a key aspect of physical function, being closely associated with cognitive function. For instance, one study on community-dwelling older adults without cognitive impairment revealed that those with slower gait speeds experienced greater cognitive decline than did those with faster gait speeds^23)^. Additionally, individuals who exhibited abnormal gait patterns, such as unsteadiness, were at higher risk for developing dementia than those with normal gait patterns (adjusted hazard ratio [aHR] 1.96). Specifically, those with abnormal gait were at a much higher risk for developing VaD than did those with normal gait (aHR 3.46)^24)^. Thus, physical function, including gait ability, has been considered a surrogate maker for cognitive decline.

We also investigated the association between physical function and cognitive decline among patients with CKD. Accordingly, we conducted a cross-sectional study examining the relationship between physical function and MCI among older patients with pre-dialysis CKD^20)^. This particular study revealed that a slow gait speed was significantly associated with the presence of MCI, even after adjusting for covariates^20)^. Furthermore, to clarify the causal relationship between physical function, kidney function, and cognitive decline, we conducted a longitudinal study over a follow-up period of 2 years^25)^. In the mentioned study, patients were classified into the following 4 groups according to various combinations of kidney and physical function values: (1) patients with mild-to-moderate kidney function impairment and high physical function; (2) those with mild-to-moderate kidney function impairment and low physical function; (3) those with severe kidney function impairment and high physical function; and (4) those with severe kidney function impairment and low physical function. Notably, our results revealed that those with both severe kidney function impairment and low physical function experienced a more significant subsequent decline in cognitive function than those with mild-to-moderate kidney function impairment and high physical function. However, no significant cognitive decline was observed in patients with high physical function, even with severe kidney function impairment^25)^. These findings therefore suggest that maintaining healthy physical function in patients with CKD may have a beneficial effect in preventing cognitive decline.

## Effects of Physical Exercise on Cognitive Function

Despite the available evidence, the actual effectiveness of physical activity and exercise in preventing cognitive decline in patients with CKD has come into question. As mentioned previously, several studies have identified factors of cognitive impairment in patients with CKD. However, strategies for preventing cognitive impairment have not been established at present. Nonetheless, recent reports have shown that exercise therapy had short-term effects on cognitive function in patients with CKD, suggesting its potential as a non-pharmacological intervention.

### Hemodialysis patients

Several reports have been available on the effects of physical exercise on cognitive function in hemodialysis patients. For example, the EXCITE trial, a multicenter randomized controlled trial (RCT) in dialysis units, reported that low-intensity intervention, consisting of approximately 20 min of walking at home for 6 months, prevented the decline in cognitive scores^26)^. Additionally, 1 meta-analysis reported that physical exercise can improve cognitive function in dialysis patients^27)^.

Nonetheless, the mentioned studies have significant limitations, making it difficult to conclusively state whether physical exercise effectively prevented cognitive decline in dialysis patients. For example, in the EXCITE trial, cognitive outcomes were measured using the cognitive function score of the Kidney Disease Quality of Life Short Form. This assessment involves answering a few questions related to cognitive function through a self-administered questionnaire^26)^. Although previous studies have reported a weak positive correlation between this assessment tool and actual cognitive function, it remains unclear whether such a tool accurately captures the patients’ cognitive functions. Additionally, the follow-up study of the EXCITE trial found no significant difference in cognitive scores between the intervention and control groups at the 18- and 36-month follow-up points^28)^. Moreover, regardless of whether a temporary improvement in cognitive function is observed, it remains unclear whether this result can prevent the future onset of dementia.

Based on the aforementioned findings, we can conclude that physical exercise may potentially offer short-term maintenance or improvement in cognitive function in dialysis patients; however, this benefit has yet to be clearly established. Furthermore, there has been no evidence to suggest that physical exercise can provide long-term maintenance of cognitive function or prevent the onset of dementia.

### Pre-dialysis CKD patients

**Fig. 3.**
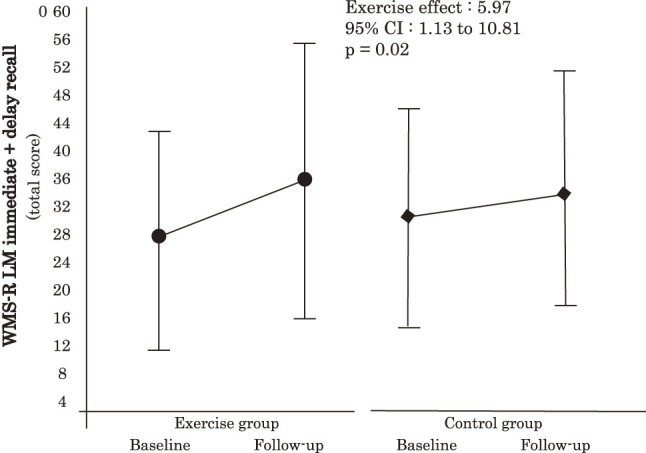
Changes in memory function, as measured by WMS-R LM immediate recall and delayed recall, in the physical exercise intervention group and control group over a 24-week period^29)^ WMS-R LM, Wechsler Memory Scale-Revised Logical Memory

We conducted an RCT to investigate the effects of physical exercise on cognitive function in pre-dialysis CKD patients^29)^. This particular study included 53 older patients with CKD aged 65 years and older (CKD stages G3–4) who were randomly assigned to an exercise intervention group (27 patients) and a control group (26 patients). Patients in the exercise group participated in group exercise training at our facility once weekly and performed independent exercises at home twice or more weekly for 24 weeks. Notably, our results showed that the exercise group showed significantly greater improvements in memory function, as measured using the Wechsler Memory Scale-Revised Logical Memory than did the control group (Fig. 3)^29)^. We believe that this result is likely due not only to the effects of the exercise intervention itself but also to the increased social interaction among patients facilitated by the group exercise sessions. However, similar to dialysis patients, the long-term effects of exercise on maintaining cognitive function and preventing dementia among pre-dialysis CKD patients still remain unclear. Therefore, further accumulation of evidence is needed in this population as well.

## Conclusion

This review provides an overview of the current states of cognitive impairment and dementia in CKD patients. With the continued aging of the patient population, there is a growing expectation for efforts to prevent cognitive impairment, particularly physical exercise and activity. However, long-term effects and specific strategies remain unclear, highlighting the need for further research and accumulation of knowledge.

## Conflicts of interest

The author declares no conflicts of interest.
